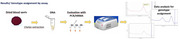# *APOE gene* screening in a cohort of Cuban late‐onset Alzheimer's disease patients using the novel diagnostic kit SUMASIGNAL APOE

**DOI:** 10.1002/alz70856_096820

**Published:** 2025-12-24

**Authors:** Yulaimy Batista Lozada, Iria García de la Rosa, Daniel Gutiérrez Luis, Yanin Mokdse Beltran, Niurka Verdecia Gorrita

**Affiliations:** ^1^ Center of ImmunoAssay, Havana, Havana, Cuba

## Abstract

**Background:**

Apolipoprotein E (ApoE) is a protein that plays a critical role in regulating lipid metabolism. It functions as a ligand for hepatic receptors and contributes to the maintenance of glucose and cholesterol homeostasis. Polymorphisms in the *APOE* gene result in the manifestation of six distinct genotypes, each with a unique function in metabolism. Genotyping facilitates the identification of individuals who are more susceptible to late‐onset Alzheimer's disease, a condition associated with the presence of allele 4 of the APOE gene. This knowledge is instrumental in guiding diagnosis, assessing risk, preventing, and treating the disease.

**Method:**

A cohort of 100 individuals, 94 of whom presented late‐onset Alzheimer's disease symptoms, was evaluated using a novel assay, SUMASIGNAL APOE. The method commenced with the extraction of genomic DNA from dried blood on filter paper using Chelex‐100 chelating resin. Allele‐specific primers were designed for the alleles and for conserved regions of the APOE gene to serve as internal controls for each amplification. Reaction mixtures Ɛ2, Ɛ3, and Ɛ4 were standardized for allelic characterization of each sample. Multiplex real‐time PCR and high‐resolution melting analysis (HRMA) were performed to distinguish alleles and internal controls.

**Result:**

The analytical method demonstrated 100% concordance with a commercial kit, intra‐ and inter‐assay coefficients of variation of less than 0.46%, and the capacity to detect up to 5 ng/µL DNA from samples per mix.

**Conclusion:**

The assay was demonstrated to be specific for the detection of target sequences and to be a simple and affordable method for determining the genotype of each individual. The development and validation of this diagnostician enabled the execution of a study, a pioneering endeavor in Cuba, that investigated allelic frequencies in a population of individuals afflicted with neurodegenerative disorders.